# Demand for cardiopulmonary resuscitation and bleeding control skills training in an urban region: a feasibility review of a community engagement effort

**DOI:** 10.3389/fpubh.2025.1339215

**Published:** 2025-03-06

**Authors:** Katherine Hoops, Ashley Bedner, Emily Kemper, Deborah Aksamit, Caitlin O’Brien, Marcie K. Johnson, Rosalyn W. Stewart, Daniella D. Maydan, Kimberly Hailey-Fair, Panagis Galiatsatos

**Affiliations:** ^1^Department of Anesthesiology and Critical Care Medicine, Johns Hopkins University School of Medicine, Baltimore, MD, United States; ^2^Johns Hopkins Bloomberg School of Public Health, Baltimore, MD, United States; ^3^Office of Cardiopulmonary Resuscitation, The Johns Hopkins Hospital, Baltimore, MD, United States; ^4^Medicine for the Greater Good, The Johns Hopkins University School of Medicine, Baltimore, MD, United States; ^5^Department of Pediatrics, The Johns Hopkins University School of Medicine, Baltimore, MD, United States; ^6^Division of Pulmonary and Critical Care Medicine, The Johns Hopkins University School of Medicine, Baltimore, MD, United States

**Keywords:** community engagement, bystander CPR, health equity, community health worker, population health, pedagogy

## Abstract

In this community engagement feasibility review, we evaluate the infrastructure warranted to implement bystander CPR and bleeding control skills training to diverse social organizations in an urban region over an 9-month period. Further, we review the necessary variables to sustain such an effort, for both the health system as well as the partnering communities. The intervention, brought forth with partnering communities through community health workers or other community brokers, for this community engagement feasibility study is the execution of teaching CPR skills and bleeding-control skills to various social networks in an urban region. The intervention focused on a changeable behavior, specifically on community members’ abilities to execute life-saving interventions. The goal is to meet the intended behavior outcome under ideal circumstances, which are training of the aforementioned skills in respective community settings over a 120-min teaching session. Over an 9-month period, we were able to implement CPR training at 5 communities reaching 136 community members. These trainings were implemented in regions that have the highest homicide rates (mean of 0.86 ± 0.14 homicides per 1,000 persons; Maryland as a state averages 0.12). Implementation of CPR and bleeding control training in diverse community settings in an urban region was feasible and cost-efficient over a 9-month period. Further, utilizing community liaisons, such as community health workers, was critical to its success.

## Introduction

The sense of community and support found in social networks is often built upon underlying ties that are embedded in the social system (1). These ties reaffirm the social cohesion and dense relationships between members, whether within a faith-based organization or tenants living in the same housing unit. Mutual connectedness allows for communities to respond to adversities together, be they due to public health crises (e.g., the COVID-19 pandemic) or more specific issues related to the network (e.g., implementation of food drives) (1–4). However, these responses often necessitate external requests by the community for the allocation of resources in an effort to disseminate information, products, or both. One of the most effective responses to these requests is through medical-community relationships (5–7), whereby a health system can react in an operational manner to assure that the appropriate resources are distributed for the community and on the community’s time line.

In the past 10 years, established, trained, volunteer community messengers, called lay health educators (8) who in Baltimore City often come from faith-based organizations, have assisted in maintaining medical-community relationships. Lay health educators are trained to provide health education and resources to strengthen health literacy in their communities. Lay health educators may also work closely with community health workers, frontline public health workers who receive additional training to help improve access to health care and reduce health disparities in addition to providing health education. These community liaisons have assisted in implementation of various health needs over the decade, with the majority of them focused on reactive health issues, from refugee crises to COVID-19 (9, 10). With ongoing trauma and violence in Baltimore City, along with media attention to increased need for cardiopulmonary resuscitation (CPR) skills (11, 12), a common and natural request from many lay health educators is training on CPR skills for their community and its respective members. Early CPR by bystanders improves survival after cardiac arrest, and bleeding control techniques such the use of tourniquets can reduce major bleeding, the leading cause of preventable death after injury. CPR and bleeding control measures save lives when administered immediately by trained responders, and given known racial disparities in provision of CPR by bystanders, providing this education must also be a priority for health systems (13). Real-time response to such a demand for training is often requested by the community due to the proximity of events that have a personal impact on the respective social network, including the daily toll of community violence. How a health system responds to such requests, in a manner that aligns with the community’s timeline and urgency, will assure the medical-community relationships are reaffirmed in their bidirectionality.

In this feasibility review, we describe our experience implementing bystander CPR and bleeding control training through established medical-community partnerships and the practicality of the deliverable. Specifically, we focus on how health systems can replicate such work in a manner that is effective and sustainable through our focus of implementation and practicality (14). Further, we examine several key insights from the communities who received the training that assist in reaffirming the value of medical-community relationships and community engagement.

## Methods

### Intervention

The intervention for this community engagement feasibility study is the execution of teaching CPR skills and bleeding-control skills to various social networks in Baltimore City with an established medical-community relationship through community health workers and/or lay health educators. The intervention focused on a changeable behavior (15)—community members’ abilities to execute life-saving interventions. The goal is to meet the intended behavior outcome under ideal circumstances, which are training of the aforementioned skills in respective community settings over a 120-min teaching session. Each participant in attendance is asked to demonstrate basic CPR skills and implement appropriate therapies for bleeding control under supervision of certified instructors. However, the goal of the session is education and skills acquisition, but no formal certification is provided.

### Implementation

To best ensure that the intervention can be fully implemented as planned and proposed in diverse community settings, from faith-based organizations to housing units to gymnasiums, locations for trainings are selected with input from established lay health educators or community health workers. Of note, community health workers are trained by the health care system’s accredited community health worker curriculum. The requirements for session implementation include selection of dates and times, availability of a sufficient number of skilled instructors and appropriate equipment, and community members present to receive the teaching. Further, we explore why the community representative requested the teaching to occur, including recent events affecting the community.

Outcomes explored include the degree of implementation, success or failure of the implementation, resources needed, and cost of implementation and necessary resources. Secondarily, barriers to and facilitators of implementation were examined.

### Practicality

With the community engagement intervention, one feasibility variable to explore is the practicality of the implementation. For this factor, an exploration to the extent the community engagement can be delivered with resources, time, and commitment by staff in the setting of constraints posed by health care institutes and the community itself. (For this factor, we explore the necessary resources, time, and commitment by staff to implement the intervention, given the constraints posed by health care institutes and the community itself.) Outcomes explored include efficiency of implementation, quality of the implementation, community feedback, and a cost analysis.

### Demographic variables

For the communities, we identified if the liaison is a lay health educator or a community health worker. Further, we will note the community’s organizational status (e.g., faith-based organization, housing unit, other), breakdown by race (percentage of Black/African Americans in the neighborhood based on the US 2020 Census) (16), and the socioeconomic status of the census tract in which the community organization resides in using the national percentiles for area deprivation index as a composite index for concentrated socioeconomic disadvantage (17). Homicide and shooting data was pulled for each community’s census tract through the Baltimore Police’s publicly available “Open Baltimore” registry for 2021 (18). The Johns Hopkins University School of Medicine Institutional Review Board (IRB) exempted this analysis.

## Results

### Communities

In 2020, the population of Baltimore City was 585,708 people according to the US Census. The Census also indicates that Baltimore neighborhoods range from 7.3 to 96.4% Black/African American. Area deprivation indices for Baltimore City range from 3 (most affluent) to 99 (most disadvantaged) based on national percentiles. Homicide rate per 1,000 residents ranges from 0 to 1.42.

The aforementioned characteristics of neighborhoods in Baltimore City should be viewed in context to the community organizations requesting CPR and bleeding control skills training. From January through September 2023, a total of five teaching events were conducted. This time period represents the nine-month pilot period during which these training sessions were first offered to the community. Two were at faith-based organizations, while three focused on youth specifically (from non-profit community youth organizations to a high school). All of the organizations were located in socioeconomically disadvantaged neighborhoods based on area deprivation index and also had among the highest rates of homicide in the city (Table 1). Table 1 has a complete list of sociodemographic variables for the organizations.

**Table 1:** Demographics of community organizations.

Organization type	Community Liaison	Area deprivation index (1–99)	Percentage of Black/African Americans in neighborhood	Homicide rate per 1,000 residents
Maryland, overall			31.7%	0.12
Community Youth Serving Group	Lay Health Educator	98	96.1%	0.68
Community Youth Serving Group	Community Health Worker	84	37.2%	0.38
High School	Lay Health Educator	98	95.4%	1.15
Faith Based Organization	Lay Health Educator	98	96.4%	1.42
Subsidized Housing	Lay Health Educator	98	96.4%	0.68

### Participants

Over this time period, we had 136 community participants. Participant and instructor demographics, including community members and health system representatives, are highlighted in Table 2. Leadership from the health system was consistent through an attending physician (pediatric critical care physician, KH) who is an American Heart Association certified instructor in Basic Life Support and Pediatric Advanced Life Support as well as an American College of Surgeons certified instructor in “Stop the Bleed” hemorrhage control training. The majority of the health system staff came from the critical care or emergency medicine fields. The aforementioned program leader assisted in identifying colleagues for participation as instructors, coordinating at times that did not interfere with competing priorities of clinical, administrative, and/or research duties. As for the community organization, the navigation of implementing the engagement was conducted through the community liaison, often a lay health educator (Table 1). These liaisons made sure the dates and times served both the health system as well as their respective community in order to assure participation by all interested community members.

**Table 2:** Participants of the medical-community engagement.

Organization type	Community participants	Health system participants		Ratio CP:HSP
		Discipline	Number	
Community Youth Serving Group	26	Pediatric Critical Care NP	2	3.3
		Pediatric Critical Care MD	4	
		Critical Care MD	1	
		ED Registered Nurse	1	
Community Youth Serving Group	19	Pediatric Critical Care NP	1	1.5
		Pediatric Critical Care MD	8	
		Critical Care MD	2	
		ED Registered Nurse	1	
		Pediatrics Resident	1	
High School	60	Pediatric Critical Care MD	3	8.6
		Injury prevention specialist	1	
		ED Registered Nurse	3	
Faith Based Organization	15	Pediatric Critical Care NP	1	2.1
		Pediatric Critical Care MD	6	
Subsidized Housing	16	Pediatric Critical Care MD	3	3.2
		Child Life Specialist	1	
		Critical Care MD	1	

CP, Community Participants; HSP, Health System Participants.

Two community engagements occurred during the weekday. One occurred in the morning of a weekday (High School), which in turn had the greatest ratio of community participants to health system participants at 8.6, which was driven due to the amount of community members that attended (60) (Table 2). The other weekday event occurred in the afternoon (subsidized housing). The remaining events occurred on weekends, with the range of the community participant to health system participant ratio ranging from 1.5 to 3.3.

In regards to time, two separate evaluations were necessary: time to plan the event and time to implement. Time to plan the event required accruing skills instructors and supplies for the health system, while for the community it meant finding space for the teaching. The community liaison’s marketing for the event was minimal (announcement in bulletins or by community leaders) as the decision to hold the event was a consensus the by community (resulting in a motivated group to attend), and therefore focused more on sharing timing and location information. The pre-event time commitment by the health system averaged 60-min or less for each event. The event time commitment by the health system averaged under 4 h: 1 hour for preparation and set up, 2 hours of active instruction, and 1 hour for breakdown and clean-up activities.

### Cost

Operating within existing CPR skills training and certification infrastructure at the health system allowed sessions to be offered at no cost to the community groups and with no additional costs to the program team. Leaders leveraged existing relationships with simulation educators and CPR educators to utilize skills curricula and available CPR mannequins, training public access automatic external defibrillators (AED), and bleeding control supplies including injury task simulators, gauze and tactical tourniquets.

With previously secured private funding support for community outreach initiatives, each site was given a high quality Stop the Bleed training kit, valued at about $250 each.

## Discussion

Implementation of CPR and bleeding control training in diverse community settings in an urban region was feasible and efficient over a 9-month period. By utilizing existing infrastructure and materials, implementation was also cost-effective, which helps to ensure that health skills promotion and dissemination by a health system can be sustainable. Time-efficiency of the intervention also facilitates participation by hospital staff to assist in the training modalities, and advance communication with the community groups helps to ensure that the sessions are adequately (but not excessively) staffed. Finally, the success of the community engagement is founded upon active medical-community relationships through designated community liaisons (either lay health educators or community health workers).

Liaisons between community organizations and healthcare systems serve a valuable role in allocating health-related resources to their respective social networks. Community health workers, for instance, have served many such purposes, from implementing access to cancer treatments to infectious disease treatments to health information (19, 20). However, different factors may influence a community’s willingness to learn and ultimately execute life-saving skills in acute situations in community settings. Our understanding of the factors that drive community willingness to learn and perform CPR, for instance, are limited, and no universal instrument has been validated to assess such variables of influence (21). The success of our implementation of community CPR and bleeding control skills despite this uncertainty was likely impacted by the efforts of the community liaisons, who (a) assured their respective community members would want to learn to the skills of bystander CPR and bleeding control; (b) coordinated with the health system in regards to planning the event and (c) confirmed community members arrived and participated on the date of training. Their deep insights into the priorities and values in their communities was critical to each session.

The correlation between the census tract homicide rates and location of the communities requesting CPR and bleeding control training warrants attention. As mentioned, there is no universal instrument validated to screen non-health-related communities for their desire to learn and implement CPR. The impetus for community liaisons to request such training likely is influenced by the proximity of traumatic events. For instance, the faith-based organization that requested training serves a community with among the highest homicide-rates in Baltimore City. Further, areas of Baltimore City with high rates of violence also had a high area deprivation index, indicating these neighborhoods are some of the most socioeconomically disadvantaged regions. These factors may influence persons who reside there to seek out skills that may provide immediate life-saving assistance. Future assessment for selecting organizations for the purpose of community engagement teaching CPR and bleeding control skills should take into account a region’s contextual variables, such as exposure to violence and socioeconomic disadvantage, as these may be motivating factors for community members and their health concerns.

One additional component to consider that warrants further evaluation is the appropriate instructor to community member ratio. Ratios that optimize training, learning, and cultural humility, while not having the perception to the community of overwhelming due to an overstaffed or understaffed group of instructors (with the latter which may strike also as insensitive to the community). Various qualitative factors should be considered in regards to this ratio. For instance, a leader that is able to communicate well to the community may offset concerns of an understaffed or overstaffed instructor group. In addition, the experience of the instructors warrants insight in regards to training and implementing teaching appropriately and effectively. Thereby, such factors may influence the ratio overall, where, for instance, a lower ratio of seasoned instructors with an effective leader implementing bystander CPR training may do well (fewer instructors, many more community members). Future qualitative studies should investigate such factors in an effort to establish the appropriate instructor to community member ratio, taking into account communication efficacy of instructor leaders and years of teaching experience by instructors, among other qualitative variables associated with such a pedagogy.

As for the feasibility of this community engagement, implementation was shown to be both cost-effective and time-efficient. Two challenges were of concern in regard to implementing and sustaining the feasibility of this community engagement project. First, provider time constraints are well documented as often impacting implementation of clinical guidelines in practice (22); therefore, additional responsibilities may be burdensome or even impossible for providers to manage. However, health system leaders sought to coordinate mutually agreeable times for both the community group and the clinician instructors with the community liaison (acting as “middle managers”) to mitigate these potential barriers (23). Furthermore, this project was initially undertaken in part as a wellness and team-building service activity for the clinician instructors, so respect for clinicians’ time and availability was critical to the success of implementation. This wellness initiative created an opportunity to provide the volunteer resources to routinely offer this training to communities, which had previously only been available on rare occasions. Second, cultural relevance of the training may impact the feasibility and sustainability, for both the health system and the community. Recruiting health instructors whose clinical experience centers on critical care including cardiac arrest and critical trauma assures that this topic is highly relevant for the health system (23, 24). As for the community, the correlation between high homicide rates and request for education on CPR skills likely is influential and reaffirms learning such skills as a culturally relevant factor. This is further evident as a diverse range of social networks requested the training sessions, indicating that this learned health skill is not exclusive to one type of organizational network and its respective mission or values.

In conclusion, implementing CPR and bleeding control skills courses in diverse community settings in an urban region was highly feasible and culturally relevant to both entities (health system and community). Various factors were identified that increased both feasibility and sustainability, as well as prioritizing cost-effectiveness and time-efficiency. Finally, designated, trained community persons (either lay health educators or community health workers) acting as liaisons between their respective social networks and the health system were key to successful engagement in this case. Moving forward, evaluating the ongoing influence of the project on the community, the health system, and whether community members utilized or felt confident to utilize their skills will be a priority for assessment in an effort to assist in the dissemination of this community engagement initiative to other health systems and regions.

## Data Availability

The raw data supporting the conclusions of this article will be made available by the authors, without undue reservation.
